# Beneficial Effects of Rosmarinic Acid In Vitro and In Vivo Models of Epileptiform Activity Induced by Pilocarpine

**DOI:** 10.3390/brainsci13020289

**Published:** 2023-02-08

**Authors:** Bruna Neuberger, Fernanda Kulinski Mello, Michele Pereira Mallmann, Karine Gabriela da Costa Sobral, Michele Rechia Fighera, Luiz Fernando Freire Royes, Ana Flávia Furian, Tuane Bazanella Sampaio, Mauro Schneider Oliveira

**Affiliations:** 1 Graduate Program in Pharmacology, Federal University of Santa Maria, Santa Maria 97105-900, Brazil; 2 Graduate Program in Biological Sciences: Toxicological Biochemistry, Federal University of Santa Maria, Santa Maria 97105-900, Brazil; 3 Graduate Program in Food Science and Technology, Federal University of Santa Maria, Santa Maria 97105-900, Brazil

**Keywords:** rosmarinic acid, seizures-like, pilocarpine, antioxidant, neuromotor damage

## Abstract

Epilepsy is characterized by a predisposition to generate recurrent and spontaneous seizures; it affects millions of people worldwide. Status epilepticus (SE) is a severe type of seizure. In this context, screening potential treatments is very important. In the present study, we evaluated the beneficial effects of rosmarinic acid (RA) in pilocarpine-induced in vitro and in vivo models of epileptiform activity. Using an in vitro model in combined entorhinal cortex–hippocampal from Wistar rats we evaluated the effects of RA (10 µg/mL) on the lactate release and a glucose fluorescent analogue, 2-(N-(7-nitrobenz-2-oxa-1,3-diazol-4-yl)amino)-2-deoxyglucose (2-NDBG), after incubation in high potassium aCSF supplemented or not with pilocarpine. In the in vivo model, *SE* was induced in male C57BL/6 mice by pilocarpine. At 1, 24, and 48 h after the end of *SE* mice were treated with RA (30 mg/kg/v.o.). We evaluated the neuromotor impairment by neuroscore tests and protein carbonyl levels in the cerebral cortex. In both in vitro models, RA was able to decrease the stimulated lactate release, while no effect on 2-NBDG uptake was found. RA has beneficial effects in models of epileptiform activity in vivo and in vitro. We found that RA treatment attenuated *SE*-induced neuromotor impairment at the 48 h timepoint. Moreover, post-*SE* treatment with RA decreased levels of protein carbonyls in the cerebral cortex of mice when compared to their vehicle-treated counterparts. Importantly, RA was effective in a model of *SE* which is relevant for the human condition. The present data add to the literature on the biological effects of RA, which could be a good candidate for add-on therapy in epilepsy.

## 1. Introduction

Epilepsy is a chronic neurological disease characterized by a predisposition to generate recurrent and spontaneous seizures with an incidence of 1–2% in the general population. In addition to seizures, the quality of life of epilepsy patients and their families is affected by several difficult-to-treat neurological comorbidities, including depression, anxiety disorders, and cognitive deficits [1]. Of importance, among the seizure forms, status epilepticus (SE) highlights itself as the most extreme type of them. Moreover, SE is an acute neurological condition causing molecular, structural, and functional reorganization, which may lead to spontaneous recurrent seizures. In this context, the pilocarpine model has been widely used and it is well established to model epilepsy in rodents. Initially, pilocarpine induces acute SE followed by a latent period, in which the chronic condition is developed for the occurrence of spontaneous recurrent seizures [2].

Understanding the molecular bases underlying seizures, epilepsy, and their comorbidities, as well as the screening of new treatments is of fundamental importance for the design of effective therapeutic strategies for preventing epilepsy or modifying the disease [3]. The study of inflammatory processes and oxidative stress and their relationship with pathologies of the central nervous system have been of great importance in research in the last decade. Indeed, experimental and clinical evidence indicates neuroinflammation and increased oxidative stress as a common and crucial constituent in epileptogenesis and the development of spontaneous recurrent seizures [2,4].

Rosmarinic acid (RA) is an ester of caffeic and 3,4-dihydroxyphenyllactic acids which was first isolated in 1958 from the rosemary plant (Rosmarinus officinalis), popularly known as rosemary, and is present in several other plant species, such as *Salvia officinalis*, and *Perilla frutescens* [5]. Interestingly, several biological activities have been described for RA [6], including antioxidant activity [7] which has been largely linked to the neuroprotective and anti-inflammatory actions of RA [8]. In the context of epilepsy, the potential beneficial effects of RA have been evaluated with discrepant results [9]. For instance, pretreatment with RA decreased seizure activity induced by kainic acid in rats [10] and by pentylenetetrazole (PTZ) and pilocarpine in mice [11]. On the other hand, RA was not able to modify PTZ-induced kindling in mice [12] or mid-term consequences of pilocarpine-induced SE in mice [11]. Nevertheless, RA may prove useful during a short time window after SE, rather than at later timepoints. Therefore, we hypothesized that RA would protect against the acute consequences of pilocarpine-induced epileptogenic events. To shed further light on the neuroprotective potential of RA, in the present study we tested the effects of RA on in vitro and in vivo paradigms of epileptic activity, in which it has not been tested to date. Specifically, we evaluated the effects of RA on neuromotor status and protein carbonylation in an acute phase (48 h) of pilocarpine-induced *SE* in mice and in an acute brain slice model for anticonvulsant screening which is based on the lactate release [13].

## 2. Material and Methods

### 2.1. Animals

Male Wistar rats aged 21 to 28 days (50 to 100 g) from the central vivarium of Federal University of Rio Grande do Sul (UFRGS, Brazil) and male C57BL/6 mice aged 30 to 60 days (25 to 30 g) from the central vivarium of Federal University of Santa Maria (UFSM, Brazil) were used. The animals were adapted to laboratory vivarium for at least 15 days before the experiments to avoid any stress due to transport and housing change. All experimental protocols aimed to limit the suffering and the number of animals used. Studies were conducted in accordance with national and international legislation (guidelines of Brazilian Council of Animal Experimentation—CONCEA—and of U.S. Public Health Service’s Policy on Humane Care and Use of Laboratory Animals), and with the approval of the institutional animal care and use committee of the Federal University of Santa Maria (approval numbers 9336090920 and 7594020715 for in vitro and in vivo studies, respectively).

### 2.2. Reagents

Rosmarinic acid was obtained from Sigma-Aldrich (product no. 536954, Sigma-Aldrich, St. Louis, MO, USA) and was dissolved in vehicle solution (0.9% NaCl containing 0.05% Tween) for in vivo testing and in artificial cerebrospinal fluid (aCSF) for in vitro studies. Pilocarpine was obtained from Sigma-Aldrich and dissolved in 0.9% NaCl for in vivo experiments and in aCSF for in vitro testing. The fluorescent glucose analogue 2-[N-(7-nitrobenz-2-oxa-1,3-diazol-4-yl)amino]-2-deoxy-D-glucose (2-NBDG) was purchased from Thermo Fisher Scientific Inc (Waltham, MA, USA).

### 2.3. In Vitro Experiments

**Figure 1:**
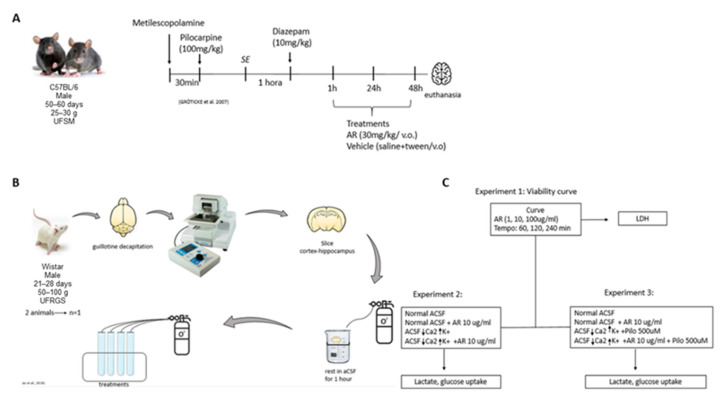
(**A**) In vivo SE induction protocol and treatments. (**B**) In vitro experimental design. (**C**) Diagram representing how the three in vitro experiments were carried out.

The experimental design is shown in Figure 1. Eighteen animals were required for all in vitro experiments. Rats were euthanized by decapitation and combined entorhinal cortex–hippocampal slices were obtained with a motorized vibratome (Campden Instruments, model 5100 mz). We decided to carry out the in vitro experiments in rats because they provide larger slices which are therefore easier to handle and maintain. Detailed protocol of sectioning is described elsewhere [14]. After sectioning, slices were allowed to rest in artificial cerebrospinal fluid (124 mM NaCl, 3 mM KCl, 1.8 mM MgSO_2_, 1.6 mM CaCl_2_, 1.25 mM NaH_2_PO_4_, 26 mM NaHCO_3_, and 10 mM glucose; 95% O_2_/5% CO_2_; pH 7.4) at 35 °C for 60 min. To ensure the results were not impacted by a loss of slice viability, we assessed time-dependent lactate dehydrogenase release (LDH) after 1, 2, and 4 h of incubation in the presence or absence of increasing concentrations of RA (1–100 µg/mL). These experiments were conducted in continuously aerated (95% O_2_/5% CO_2_) glass tubes containing aCSF. At appropriate timepoints, one slice and 1 mL of the incubation medium were collected for analysis of LDH release. Concentrations of RA used in the present study were selected based on the literature [15] and on pilot concentration–response experiments.

The effect of RA (10 µg/mL) on lactate release [13] and glucose uptake [14] was performed in two models of in vitro epileptiform activity. The models were established by altering the composition of the aCSF. In the high concentration of K^+^ model, KCl was supplemented to achieve a final K^+^ concentration of 7.5 mM [16]. In the pilocarpine model the high K^+^ aCSF was supplemented with 50 µM pilocarpine [17]. The incubation time of the slices in these experiments was 4 h as chosen based on the time–response curves. RA was always added at the beginning of incubation. At the end of incubation, the slices and incubation media were collected and processed for analysis of LDH release, lactate release, and 2-NBDG uptake [14].

#### 2.3.1. LDH Release

LDH release was measured using commercially available kits (Labtest, Lagoa Santa, MG, Brazil) following the manufacturer’s instructions. The total content of LDH in samples was considered as the sum of LDH in slices plus incubation media and was set as 100% for calculation purposes. LDH release in the media was expressed as percent values of total LDH content.

#### 2.3.2. Lactate Release

Lactate release in the incubation media was demonstrated by rapid assays for seizure-like activity in brain slice models for staged anticonvulsant screening [13]. Lactate content was measured using commercially available kits (Labtest, Lagoa Santa, MG, Brazil) following the manufacturer’s instructions.

#### 2.3.3. Glucose Uptake

Glucose uptake was measured on slices using the fluorescent glucose analogue 2-NBDG according to [14]. After the end of the incubation, one slice of each treatment was carefully relocated to a tube containing aCSF supplemented with 2-NBDG (30 µM) and maintained in the media for 15 min. Following 2-NBDG uptake, the slices were washed with aCSF and homogenized in 30 mM Tris-HCl (pH 7.4). Homogenates were centrifuged at 3000× *g* for 10 min and supernatant fluorescence was measured in FlexStation 3 Multi-Mode Microplate Reader (Molecular Devices, Sunnyvale, CA, USA). One 2-NBDG uptake sample from experiments with K^+^ and pilocarpine is missing because it was lost during processing.

### 2.4. In Vivo Experiments

Twenty-two animals were required for in vivo experiments. SE was induced in 14 C57BL/6 mice following standard protocol [18] where repeated low doses of pilocarpine (100 mg/kg, ip) are injected every 20 min until SE onset. To avoid peripheral cholinergic effects, methylscopolamine (1 mg/kg, i.p.) was administered 30 min before the application of pilocarpine. Three animals did not survive SE. Sixty minutes thereafter SE was quelled with diazepam (10 mg/kg, i.p.). We decided to carry out the in vivo experiments in mice to allow a more straightforward comparison with our previous studies [11]. After 60 min of SE, we started the treatment with RA (30 mg/kg, v.o.). Animals received 3 doses of RA: 1 h, 24 h, and 48 h after SE [11]. One hour after the last treatment mice were euthanized and the cerebral cortex was removed for analysis of protein carbonyl content. Control animals went through the same protocols but received vehicle instead of other drugs.

#### 2.4.1. Neuroscore

Neuromotor impairment was assessed using the neuroscore (NS) method [19]. Animals were scored according to the degree of neuromotor impairment (0 = severely impaired to 4 = normal). First, the animals were placed on a grid to measure how many times they missed walking. Forelimb function was assessed by observing whether the animals gripped the cage when lifted and brought towards it. Afterwards, the animals were suspended, and their ability to extend their lower limbs was noted. Finally, the animals were tested for right and left lateral drive to determine resistance. This assessment was performed before SE induction to establish a baseline measure and at 24 h and 48 h after SE.

#### 2.4.2. Dot Blot Assay

Levels of protein carbonyls, a widely used oxidative-stress-induced protein damage marker, were measured in the cerebral cortex by a standard dot blot method [20]. Dot blot assay yields identical results to Western blot regarding total protein carbonylation, and it is simpler to carry out [21]. Brain samples were homogenized 1:10 (*m*/*v*) in RIPA buffer (Sigma-Aldrich product # R0278) supplemented with a cocktail of protease inhibitors (Sigma-Aldrich product # P83401) and centrifuged at 13,200× *g* at 4 °C for 20 min and supernatants were collected. An aliquot of the supernatants was incubated at room temperature for 20 min with 10 mM 2,4-dinitrophenylhydrazine (freshly prepared in 2 N HCl). The samples were neutralized with neutralization solution (2 M Tris in 30% glycerol) and then applied to a nitrocellulose membrane through a vacuum system on Dot blot equipment (Bio-Rad). Membranes were blocked with a 5% (*w*/*v*) non-fat dry milk for 1 h at room temperature and exposed overnight at 4 °C to primary antibodies raised against DNP (1:1000, Sigma-Aldrich product # D9656). Membranes were washed three times with TBS-T (0.04% (*v*/*v*) Tween 20) and exposed to anti-rabbit secondary antibodies (1:10,000, Sigma-Aldrich product # A0545) during 1 h at room temperature. Membranes were washed three more times with TBS-T and immunoreactivity was detected in a Chemidoc imaging system (Bio-Rad) using a luminol-based ECL substrate (Bio-Rad product #1705060). Signals were quantified using ImageLab v6.0 (Bio-Rad). Data from vehicle-treated controls were averaged and considered as 100%.

#### 2.4.3. Protein Content

Protein content was measured using Sigma-Aldrich’s Bicinchoninic Acid (BCA) Protein Assay Kit (product #BCA1) as per manufacturer’s instructions. Briefly, BCA’s principle is based on the formation of a Cu^2+^-protein complex, followed by Cu^2+^ to Cu^1+^ reduction. The protein content is proportional to Cu reduction, which forms a purple-blue complex detected spectrophotometrically at 562 nm. Bovine serum albumin (1 mg/mL) was used to develop the standard curves.

### 2.5. Statistical Analyses

Slice viability (LDH release) was evaluated by two-way ANOVA (time of incubation × incubation media) followed by Newman–Keuls post hoc test. Lactate release and 2-NBDG uptake were evaluated by one-way ANOVA followed by a Newman–Keuls post hoc test. Latency to SE was analyzed using unpaired Student’s t test. Parameters obtained from in vivo experiments (NS and protein carbonyls) were evaluated by two-way ANOVA followed by Newman–Keuls post hoc test. *p* values smaller than 0.05 were considered statistically significant.

## 3. Results

### 3.1. In Vitro Results

**Figure 2:**
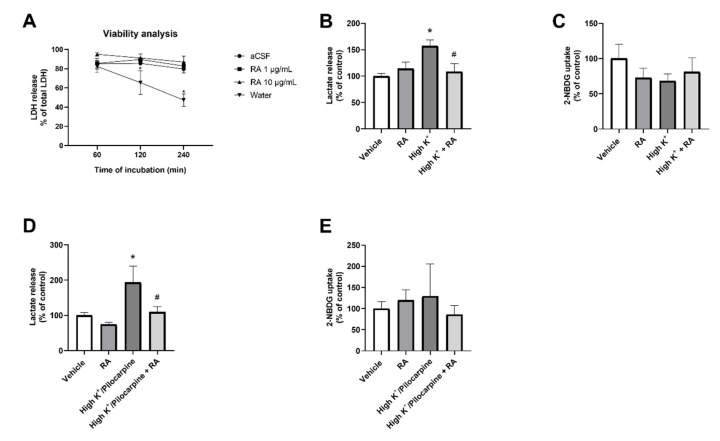
(**A**) Concentration–response and time–response experiments aimed to check viability of slices. In this experiment, slices were incubated in continuously aerated aCSF, in the presence or absence of RA. Continuous incubation in water served as negative control of viability. The asterisk (*) indicates a statistically significant difference (*p* < 0.05) when compared to the other conditions at the same timepoint. Data are presented as mean with standard error of mean for *n* = 5 per group. Effect of high concentration of K^+^ or RA (10 µg/mL) on lactate release (**B**, *n* = 7) or 2-NBDG uptake (**C**, *n* = 7) in combined entorhinal cortex–hippocampal slices from rats. Data are presented as mean with standard error of mean. The asterisk (*) indicates a statistically significant difference (*p* < 0.05) when compared to control aCSF. The hashtag (#) indicates a statistically significant difference (*p* < 0.05) when compared to RA-supplemented epileptogenic media. Effect of high concentration of K^+^ plus pilocarpine or RA (10 µg/mL) on lactate release (**D**, *n* = 6) or 2-NBDG uptake (**E**, *n* = 5) in combined entorhinal cortex–hippocampal slices from rats. Data are presented as mean with standard error of mean. The asterisk (*) indicates a statistically significant difference (*p* < 0.05) when compared to control aCSF. The hashtag (#) indicates a statistically significant difference (*p* < 0.05) when compared to RA-supplemented epileptogenic media.

To assess the time course of sample viability in our conditions, slices were incubated in continuously aerated aCSF in the presence or absence of RA (Figure 2A). The viability was maintained stable at high values for at least 240 min, except for slices incubated in water (negative control) instead of aCSF [F(3,48) = 11.93; *p* < 0.05]. Therefore, subsequent experiments were performed at the longer timepoint (240 min). RA (1 or 10 µg/mL) did not impact slice viability in the time window analyzed. However, we noticed that the highest concentration of RA (100 µg/mL) had strong interference in LDH readings, precluding the use of that concentration of RA in our experimental conditions.

The effect of RA on lactate release and 2-NBDG after slice incubation in the high concentration of K^+^ model is shown in Figure 2B–E. Lactate levels increased ~1.6-fold on average in the media supplemented with a high concentration of K^+^, and co-incubation with RA (10 µg/mL) attenuated the rise in lactate levels (Figure 2B) [F(3,24) = 4.735; *p* < 0.05]. On the other hand, no effect of high concentration of K^+^, RA, or their combination was found in 2-NBDG uptake (Figure 2C) [F(3,24) = 0.7356; *p* > 0.05].

In the last set of in vitro experiments, a high concentration of K^+^ plus pilocarpine was employed. Lactate levels increased ~2.2-fold on average in the incubation media, suggesting that pilocarpine had a facilitatory effect on lactate release. Interestingly, co-incubation with RA blunted the increase in lactate levels in the incubation media supplemented with K^+^ and pilocarpine (Figure 2D) [F(3,20) = 4.116; *p* < 0.05], suggesting RA was effective even considering the higher lactate release potential of the pilocarpine-containing media. Once again, 2-NBDG uptake was not altered by the epileptogenic media, RA, or their combination (Figure 2E) [F(3,16) = 0.2111; *p* > 0.05].

### 3.2. In Vivo Studies

**Figure 3:**
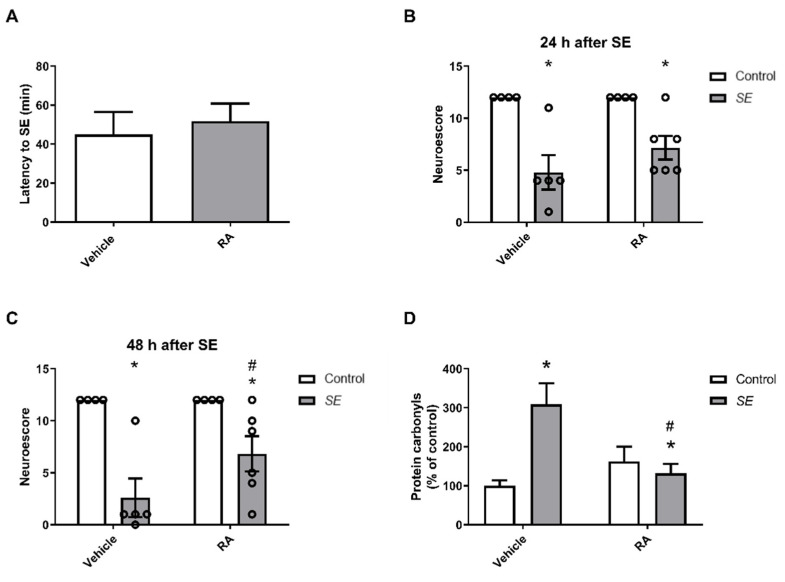
(**A**) Mice were allocated into experimental groups according to the latency to SE onset. SE was performed in three independent experiments, carried out on different days. During the experiments, unpaired Student’s t-test was applied to guide rat allocation into experimental groups. (**B**) Effect of RA (30 mg/kg; v.o.) on mice neuromotor performance (NS test) 24 h after SE. (**C**) Effect of RA (30 mg/kg; v.o.) on mice neuromotor performance (NS test) 48 h after SE. Raw NS data (individual scores) are shown as open circles. (**D**) Protein carbonyl content in the cerebral cortex of mice 48 h after SE. The asterisk (*) indicates a statistically significant difference (*p* < 0.05) when compared to vehicle-treated control animals. The hashtag (#) indicates a statistically significant difference (*p* < 0.05) when compared to RA-treated SE animals. Data are presented as mean with standard error of mean for *n* = 4–6 per group.

The latency for SE in both groups that received pilocarpine is shown in Figure 3A. Statistical analysis showed that there was no significant difference [t(9) = 0.4744; *p* > 0.05] between the groups, so they can be considered equal from the point of view of sensitivity to SE.

When analyzing the NS we found that pilocarpine-induced SE impaired neuromotor abilities at 24 [F(1,15) = 279.2; *p* < 0.05—Figure 3B] or 48 [F(1,15) = 23.54; *p* < 0.05—Figure 3C] hours after SE, and we found that pilocarpine-induced SE impaired neuromotor abilities, as they show a worsening in the test performance. Interestingly, post hoc analyses indicated that RA-treated animals had higher NS values after SE than their vehicle-treated counterparts (*p* < 0.05), suggesting that RA improved neuromotor impairment in the acute phase of pilocarpine-induced SE.

The results of the carbonyl protein measurements are shown in Figure 3D. Levels of the oxidative stress indicator increased in the animals that underwent SE, presenting a significant difference when compared to control animals [F(1,15) = 10.22; *p* < 0.05]. Importantly, treatment with RA attenuated the SE-elicited oxidative damage to proteins, since post hoc analyses revealed significant differences between the RA- and vehicle-treated SE groups (*p* < 0.05).

## 4. Discussion

Several biological activities have been described for rosmarinic acid, such as significant neuroprotective and neuroregenerative effects in various nervous system disorders [6]. A previous study by our group [11] showed that acute treatment with RA was able to increase the latency for myoclonic seizures induced by PTZ or pilocarpine in mice. However, no effect of RA was detected on mid-term (14 days) consequences of pilocarpine-induced SE. In the present study, we used the same treatment dosing and schedule [11], but we focused on short-term (48 h) consequences of pilocarpine-induced SE. Our results revealed that the treatment with RA promoted an improvement in the animals’ neuromotor performance, as measured by the neuroscore test. Moreover, we found a decrease in the level of protein carbonylation after SE in RA-treated animals, indicating that the treatment was able to reduce oxidative damage in the cerebral cortex of these animals.

These results agree to some extent with previous studies using rosemary (*Rosmarinus officinalis* L.) extracts or pure RA [22]. For instance, treatment with rosemary extract protected against kainic-acid-induced spatial memory impairment neuronal degeneration, probably because of its antioxidant properties [23]. In addition, treatment with RA decreased kainic acid seizure intensity, subsequent apoptosis, hippocampal mossy fiber sprouting, and oxidative stress markers [10]. Altogether, these and our present results are of importance since SE is a medical emergency with high potential of morbidity and mortality and that is often very difficult to treat. In this context, the pilocarpine-induced SE is an animal model capable of simulating many features of human SE and temporal lobe epilepsy depending on the timepoint employed for analysis for behavioral and neurochemical parameters [2]. Regarding this point, SE is a very drastic event, and such widespread seizure activity causes brain oxidative stress [4].

Therefore, therapies that minimize oxidative stress and neuronal damage caused by SE have been sought [24]. Furthermore, treatments targeting oxidative stress may not only be neuroprotective after SE, but also antiepileptogenic [25]. Gorter et al. demonstrated that several genes involved in oxidative stress were upregulated in the hippocampal CA3 area and entorhinal cortex during both acute and latent phases of epileptogenesis [26]. For example, *fos oncogene* (*Fos*) was acutely activated, displaying an increase of approximately 30-fold in the CA3 area, while superoxide dismutase 2 (*Sod2*) remained elevated in the chronic phase [26]. Of note, immune and inflammatory responses—crucial processes in the acute and long-term sequelae of SE—are closely linked to oxidative stress [27]. In fact, novel treatment approaches targeting oxidative and inflammatory pathways have demonstrated clinically relevant disease-modification effects [28,29,30,31]. In this way, since RA presents beneficial effects on neuromotor disruption and oxidative damage, our present results corroborate the idea that antioxidant drugs prevent sequelae in animals developing epilepsy when administered during *SE* and transiently after that.

To further investigate the beneficial effects of RA on epileptiform activity we used two in vitro models. These have been increasingly valuable to the study of the mechanisms involved in seizures, as well as the potential for new therapeutic approaches [32]. In the present study, we found an increase in lactate levels that were reduced when treated with RA. These data seem interesting since lactate has been considered a useful seizure-like activity biomarker, as demonstrated by an increase in its levels in the extracellular environment of the human hippocampus during seizures [33]. Moreover, measurement of lactate release in incubation media of organotypic hippocampal cultures has been shown to be an excellent strategy in anticonvulsant screening, allowing for a high-throughput search of potential new drugs with antiseizure activity [13]. Despite its role as an energy substrate and biomarker of seizure activity, recent studies have suggested that lactate acts as a signaling molecule in the brain [34]. For instance, it has been reported that lactate increases neuronal activity through mechanisms involving NMDA receptors, Erk1/2 phosphorylation, and Ca^2+^ influx [34]. In this context, the ability of RA to prevent lactate increase in epileptogenic media may represent not only the mitigation of a seizure-like activity biomarker, but also a mechanism underlying the anticonvulsant and neuroprotective effects. Nevertheless, this interesting possibility has yet to be investigated. In fact, the extracellular concentration of L-lactate in the brain rapidly changes between the micromolar and millimolar range under pathologic conditions such as seizures and ischemia [34]. As noted above, an increase in the extracellular levels of L-lactate may well contribute to the maintenance of seizure activity through potentiation of NMDA receptor activity and increased intracellular calcium concentration. Therefore, preventing the increase in L-lactate levels may attenuate these excitatory factors, leading to anticonvulsant activity. Since RA was able to prevent the increase in the L-lactate levels in slices continuously incubated with pilocarpine/high K^+^, it is possible that this event may underlie some of the beneficial effects of RA in pathological conditions.

One last point to discuss involves the experiments with the fluorescent glucose analog 2-NBDG. Previous studies have shown glucose hypometabolism in the brain of epilepsy patients [35] and in the brain of rats in the pilocarpine model of epilepsy [14,36]. While glucose hypometabolism may be explained by the neuronal loss occurring in chronic epilepsy, it may also represent a mechanism underlying the development and maintenance of an epileptic network (Zhang et al., 2015). On the other hand, it has been shown that the increased uptake of 2-NBDG correlates well with acute epileptic activity induced in vitro by low magnesium plus picrotoxin [37] or in vivo by 4-aminopyridine [38]. However, in the present study, none of the two in vitro models tested altered the uptake of the fluorescent glucose analog 2-NBDG, suggesting that glucose uptake by slices was not affected by epileptiform activity in our experimental conditions. One possible explanation is that longer times of incubation with 2-NBDG may be required to demonstrate increased uptake in brain slices. Alternatively, the use of more sensitive methods, such as confocal laser scanning microscopy, may reveal subtle or localized differences in glucose uptake during acute epileptogenic events.

## 5. Conclusions

In summary, our results suggest that RA can attenuate the acute neuromotor disruption and oxidative damage caused by SE in mice and has beneficial effects in in vitro models of epileptiform activity as measured by lactate release. Importantly, RA was effective in a model of SE which is relevant for the human condition. Thus, RA can be considered a promising candidate for add-on treatment with anticonvulsant drugs, although more studies are needed to define the full potential of this naturally occurring molecule.

## Data Availability

All the relevant data generated and analyzed during the current study are available upon reasonable request to the corresponding author.
